# Cardioprotective Effect of *Rumex vesicarius* Linn. Leaf Extract against Catecholamine-Induced Cardiotoxicity

**DOI:** 10.3390/molecules27113383

**Published:** 2022-05-24

**Authors:** Imran Ahmad Khan, Musaddique Hussain, Nadia Hussain, Ali M. Alqahtani, Taha Alqahtani

**Affiliations:** 1 Department of Pharmacology, The Islamia University of Bahawalpur, Bahwalpur 63100, Pakistan; 2 Department of Pharmaceutical Sciences, College of Pharmacy, Al Ain University, Al Ain 64141, United Arab Emirates; nadia.hussain@aau.ac.ae; 3 Department of Pharmacology, College of Pharmacy, King Khalid University, Abha 62529, Saudi Arabia; amsfr@kku.edu.sa (A.M.A.); ttaha@kku.edu.sa (T.A.)

**Keywords:** LDH, adrenaline, *Rumex vesicarius*, cardioprotective, troponin

## Abstract

*Rumex vesicarius* (L.) is a folklore medicinal herb that has been used for centuries to cure cardiovascular diseases. The present work was carefully designed to ascertain the pharmacological basis for *R. vesicarius*’s therapeutic efficacy in cardiovascular diseases, as well as the underlying mechanism. In the ex vivo investigation, the aqueous-methanolic leaf extract of *R. vesicarius* was shown to have endothelium-dependent vasorelaxant effects in rabbit aorta tissue preparations, and its hypotensive responses were quantified by pressure and force transducers coupled to the Power Lab Data Acquisition System. Furthermore, when rabbits were subjected to adrenaline-induced myocardial infarction, *R. vesicarius* demonstrated cardioprotective characteristics. In contrast to the intoxicated group, the myocardial infarction model showed lower ALP, CK-MB, CRP, LDH, ALT, troponin, and AST levels (*p* > 0.005–0.000), as well as edema, necrosis, apoptosis, inflammatory cell enrolment, and necrosis. *R. vesicarius* exhibited significant antioxidant activity and delayed noradrenaline-induced platelet aggregation. Its cardioprotective, anticoagulant, and vasorelaxant properties in both investigations (in vivo and ex vivo) are mediated through partial endothelium-dependent, NO and calcium channel blockade mediated vasorelaxation. The minimizing of adrenaline, oxidative stress, and tissue damage demonstrate its therapeutic efficacy in cardiovascular diseases.

## 1. Introduction

Cardiovascular disease (CVD) continues to grow in prominence as the most significant contributor to death. The epidemiological transition of the twentieth century established cardiovascular disease as the leading cause of global disability. Global health predictions indicate that it will continue to be the leading cause of death in 2030 [1]. CVD and associated atherothrombotic consequences result from abrupt changes in lifestyle, environmental variables, and genetic predisposition. CVD is a collection of heart and blood vessel disorders. It includes coronary heart disease (CHD), peripheral arterial disease (PAD), various kinds of angina, congestive heart failure (CHF), and myocardial infarction (MI) [1]. Hypertension is the leading cause of mortality in humans [2]. According to research, people in the South Asian region are more likely to suffer from cardiovascular disease (CVD) than their counterparts in other parts of the world.

Numerous developing countries, including Pakistan, Bangladesh, Iran, Afghanistan, and India, are rapidly catching up to this epidemic due to dramatically altered lifestyles [2]. Fast food, additives, preservatives, genetically modified foods, and a sedentary lifestyle all contribute to the suffering of cardiovascular diseases [3]. While synthetic medications are very successful in treating cardiovascular disorders, their utility is limited as a result of their adverse effects [4]. MI is a prevalent ischemic disease caused by extensive myocardial tissue damage. It develops due to an imbalance between oxygen demand and blood supply in cardiomyocytes with or without atherosclerotic plaque [5]. Excessive generation of reactive oxygen species (ROS) is responsible for endocardial lipid peroxidation. Such damage leads to the loss of cardioprotective antioxidants, an increase in oxidative stress, and apoptosis [5]. Likewise, catecholamine has an inotropic and chronotropic effect on the heart. Excessive catecholamine results in coronary vasoconstriction, increasing myocardial oxygen demand and decreasing myocardial blood supply, leading to MI [6].

Adrenaline (ADR) is a catecholamine secreted by the medullary portion of the adrenal glands. ADR is a non-selective agonist for all adrenergic receptors, including alpha, beta, and corresponding subtypes, principally located in the cardiovascular system [7]. Adrenaline hormone is secreted by the adrenal gland to manage the stress that increases heart rate, improves the force of the heart’s contraction and cardiac output, raises blood pressure, dilates the bronchioles in the lungs, and raises glucose and cholesterol levels in the blood. Adrenaline secretion is a component of the human “fight or flight” reaction, a rapid response to fear, potential threat, or panic. ADR is a medication that has been authorized for human cardiopulmonary resuscitation.

Additionally, it is used therapeutically to treat allergic responses, glaucoma, asthma, and cardiopulmonary arrest [8]. Doses higher than the therapeutic dose (2 mg/kg b.w.) can induce MI [5]. This model was used to estimate the cardioprotective impact of test medicines in a laboratory animal model of MI. MI is caused by lipid peroxidation (LPO), resulting in the loss of intracellular antioxidants [6]. It results in an increase in the formation of nitrosative derivatives, which results in excess of reactive oxygen species (ROS). As a result, the myocardium is subjected to severe oxidative stress [9]. It elevates Ca^++^ levels by activating calcium channels (L-type) on cardiomyocytes and enhances oxidative stress through increased workload [7]. At higher doses, it causes vasoconstriction of the aorta and coronary arteries. Additionally, it increases platelet count, clotting factor VIII, and fibrinolysis [5].

Medicinal herbs are effective in treating a wide variety of disorders worldwide. Herbal medications play a critical role in both conventional and contemporary therapies. These herbs are widely available and considered less hazardous than synthetic pharmaceuticals [10]. Their variety in phytoconstituents and their complementary and synergistic effect by down or up-regulation of various biochemical pathways adds extra charm.

*Rumex vesicarius*, Linn. (Polygonaceae) is native to Saudi Arabia, India, Iran, Pakistan, and Bangladesh and cultivated in other parts of the world. Known as ‘Chuka, Chukar, and Khatpalak’ in native languages [11], it is considered an excellent food plant with well-studied medicinal importance. Extract prepared from various parts of the *R. vesicarius* have been utilized in Indian Ayurvedic, Chinese herbal, and traditional and folk medicines for several centuries to treat emesis and diarrhea [12]. HPLC analysis revealed so many phytoconstituents such as ß-carotenes, luteolin, epicatechin, chromone, apigenin, quercetin, rutin, endocrine, catechin, emodin, physcion, chrysophanol, vitamin C, proteins, lipids, and organic acids and minerals; Fe, Na, Cu, K, Mg, Ca and Mn [13]. In indigenous medicine, the seeds’ decoction has been used to treat fever, venereal illness, rheumatism, and leprosy [11]. Bacterial infections, rheumatic aches, and malignancies are treated with leaf extract [12]. Pharmacological studies reported its antioxidant, analgesic, lipid-lowering, antimicrobial, anti-obesity, insecticidal, antipyretic, gastroprotective, anticancer, and antidiabetic effects [14]. It was devised to address cardiac diseases in folk medicine [15,16,17,18]. Plants recognized for hepatoprotection, antioxidants, antidiabetic, antithrombotic, and anti-inflammatory, are believed to provide cardioprotection. Previously we have reported antipyretic, antiemetic, antidiarrheal, bronchodilator, wound healing, and counter-irritant potential of *R. vesicarius* [16,17,18,19,20,21]. There has been no published pharmacological rationale regarding its use in cardiovascular diseases. The present study (in vivo, in vitro and ex vivo) was conducted to evaluate the hypotensive, cardioprotective, anticoagulant, and antioxidant effect of *R. vesicarius* leaf extract (aqueous-methanolic) in rabbits. We decided to employ rabbits as an animal model rather than rats and mice since their characteristics are more akin to those of human beings. Compared to rats, the calcium channels in rabbits’ aorta have a greater variety of functions [1,5].

## 2. Materials and Methods

### 2.1. Plant Materials

*R. vesicarius* (fresh leaves) were obtained from agricultural lands in Mondka, Muzaffargarh district. A professional botanist confirmed it at the IPAB, BZ, University, Multan. The voucher specimen (F.P.ST-215) has been amassed for future reference.

### 2.2. Extract Preparation

Fresh leaves were collected and subjected to shade drying. The waste (vegetative and adulterants) were removed manually. A special herbal grinder was used to make the dried leaves into a coarse powder. To retain the uniformity of the extract, powdered leaves from only one batch were used. *R. vesicarius* leaf powder (250 g) was set to soak in an aqueous-methanolic solvent (70:30 *v*/*v*) for 9 days. Amber-colored air-tight lab glass bottles were used for the maceration. Muslin cloth and Whatman-1 filter paper were used to filter the socked material. A rotary evaporator was used for the evaporation under reduced pressure at 37 °C [22]. With the help of this formula, the percent yield was calculated using this formula (Equation (1)):

(1) $$\%\mathrm{age}\mathrm{yield}=\frac{\mathrm{Theoretical}\mathrm{Yield}\left(g\right)}{\mathrm{Actual}\mathrm{Yield}\left(g\right)}\times 100$$

### 2.3. Animals

Local bread male rabbits with an average weight of 1.5 kg were obtained from the animal house of the Department of Pharmacology. They were fed on commercially available A-grade feed and tap water ad libitum. The temperature was kept at 27 °C. All tests were conducted following NIH rules for animal use [23]. The experimental design was validated by the relevant committee of the Department of Pharmacology (AS and RB/10/8/20).

### 2.4. Chemicals

Verapamil, phenylephrine, and heparin were purchased from Pfizer Laboratories, KHI, SD, Pakistan (PVT.) Ltd. Methanol was purchased from Lahore Laboratory Chemicals, Lahore, PB, Pakistan. Adrenaline was purchased from Barrett Hodgson, KHI, SD, Pakistan. ALP, ALT, LDH, CRP, troponin, AST, and CK-MB Kits were obtained from Shalimar Scientific Stores, Rawalpindi, PB, Pakistan. All solutions, chemicals, and reagents used in the experiments were standard purity.

### 2.5. Preliminary Phytochemical Investigation

Phytochemistry was conducted to detect the presence/absence of numerous phytochemical classes in the *R. vesicarius* aqueous-methanolic leaf extract, such as glycosides, alkaloids, tannins, saponins, anthraquinones, and flavonoids [24].

### 2.6. HPLC Analysis

**Figure 1:**
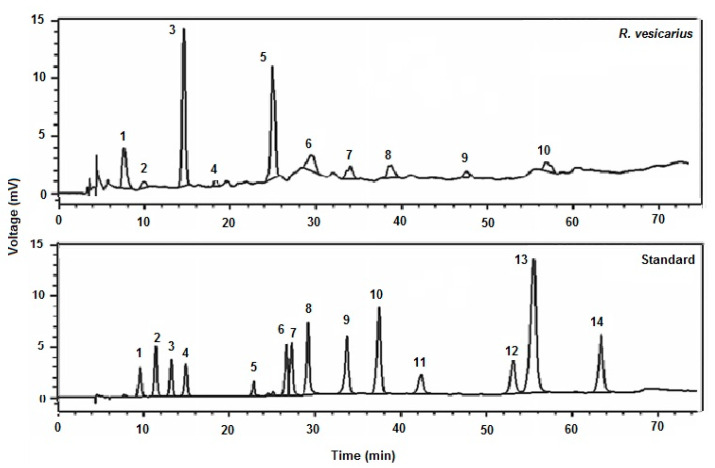
HPLC chromatogram of *R. vesicarius* extract showing the phenolic profile (kaempferol, gallic acid, catechin, coumarin, *p*-coumaric acid, ferulic acid, emodine, rutin and quercetin), concerning retention time of standard.

The phenolic acids in *R. vesicarius* leaf extract (aqueous-methanolic) were quantified by HPLC [13]. In HPLC, a binary gradient solvent system was established in conjunction with a C-18 column with dimensions (250 4.6 mm), which was able to separate 8–9 phenolics in 36 min at a flow rate of 0.0008 L/min and a film thickness of 5 µm, with the oven set to 30 °C. The separation of components was reproducible using (run-to-run), kaempferol, gallic acid, catechin, coumarin, *p*-coumaric acid, ferulic acid, emodine, rutin and quercetin were prepared as standards (purity > 99 percent), purchased from Aldrich (St. Louis, MO, USA), and the dilutions were prepared with methanol to attain a concentration of 50 g/mL. The samples were identified by comparing the retention periods of *R. vesicarius* samples to industry standards. The separation factor and resolution were utilized to determine the efficiency of HPLC separations, as shown in Figure 1.

### 2.7. Acute Oral Toxicity Dose Test

Tests were conducted on 18 rabbits to evaluate how much *R. vesicarius* was toxic to them. They were split into three groups of six rabbits each, and they were deprived of feed for 24 h before getting doses of 1000, 3000, and 6000 mg/kg orally. For 14 days of taking the *R. vesicarius* the rabbits were inspected for jerkiness, tiredness, and death [25].

### 2.8. Determination of DPPH Assay

The DPPH test was performed as described previously [5]. In brief, various concentrations of *R. vesicarius* aqueous-methanolic leaf extract (4 mL) were added to the DPPH solution and diluted to 5 mL with methanol. Then this solution was incubated in the dark for 40 min. A spectrophotometer was used to determine the absorbance of the specified solution at 517 nm. All studies were repeated thrice, and the percent inhibition in vitamin C equivalents was calculated. With the help of this formula (Equation (2)), the percent DPPH scavenging potential was calculated:

(2) $$1\%=\;\frac{A\left(\mathrm{blank}\right)-B\left(\mathrm{sample}\right)}{A\left(\mathrm{blank}\right)}\times 100$$

### 2.9. Nitric Radical Scavenging Assay

The extract was formulated from aqueous-methanolic leaf extract at a concentration of 10 mg/mL. The extract was then repeatedly diluted with distilled water to produce concentrations ranging from 100 to 1000 μg/mL, and the same process was applied to standard Gallic acid. Solutions were kept at 4 °C for the experimentation. Freshly prepared Griess reagent was used for the reaction. A volume of 0.5 mL of 10 mM sodium nitroprusside in phosphate buffered saline was mixed with 1 mL of each of the extract strengths (100–1000 μg/mL) and incubated at 25 °C for 3 h. An equal volume of freshly produced Griess reagent was added to the extract. Control samples were made in the same way as the test samples, but without the extracts and with an equivalent volume of buffer. The color tubes contained the same quantities of extracts but no sodium nitroprusside. The reaction mixture was transferred to a 96-well plate in a volume of 150 μL. A UV-Vis microplate reader was used to measure absorbance at 546 nm (Alibaba, Hangzhou, China) as described in our previous communications [5,6]. The extract and standard’s percentage inhibition was computed and recorded. With the help of the following formula (Equation (3)), extracts and Gallic acid’s percentage nitrite radical scavenging activity was estimated;

% nitrite radical scavenging activity: A − B/A × 100,(3)

where A = absorbance of control sample and B = absorbance in the presence of extracts or standards samples.

### 2.10. Acute Myocardial Infarction Study

The animals were divided into five groups, each with six animals. For two consecutive days, the rabbits in Group 1 received normal saline, whereas the animals in Group 2 received ADR (2 mg/kg b.w. Sc) every 24 h of a gap. Group 3, Group 4, and Group 5 rabbits received 100, 200, and 300 mg/kg *R. vesicarius* extract by oral gavage for 14 consecutive days before receiving ADR 2 mg/kg on the 14th and 15th days, separated by 24 h, respectively [26]. The three doses were chosen based on earlier published studies on the hepatoprotective properties of *R. vesicarius* [27]. On the sixteenth day, blood samples were collected from the marginal ear veins of rabbits to determine the levels of biochemical markers such as ALT, troponin, ALP, CK-MB, CRP, LDH, and AST in blood using standard kits.

#### Screening of Cardiac Weight to Body Weight Ratio

The ratio of cardiac weight to bodyweight assists in determining the cardiac weight index and necroses [1,6].

### 2.11. Histopathology

Under general anesthesia, rabbits were euthanized, and the hearts were dissected for histological study. The ventricular part of the heart was rapidly transferred to a 10% formalin solution. The tissue was then submerged with paraffin. Before mounting in xylene, a 5-m μm piece was cut and stained with hematoxylin-eosin [28]. Microscopical examination of the ventricular area of cardiac tissue from several groups was used to assess the ADR effect on the cellular architecture of the heart alone and in conjunction with three groups treated with *R. vesicarius*. Micro-images were captured using a compound microscope connected to a camera LCD.

### 2.12. Vasorelaxant Activity

After slaughtering the rabbits, the thoracic cavity was excavated, and the aorta was meticulously dissected. The aorta’s fat and connective components were cautiously removed in a Petri dish containing Krebs’ solution. Aortic rings (3–4 mm in length) were sliced and put in a tissue organ bath (10 mL) already having the Krebs–Henseleit solution and being bubbled continuously with carbenogen gas (95% O_2_ and 5% CO_2_). The lower hook was locked in place, while the upper was linked to a force-displacement transducer connected to a Powerlab data collection device to trace the isometric contractions. Throughout the 40-min stabilization phase at 1.0 g resting tension, Kreb’s solution was replenished every 15 min to avoid metabolite buildup. Following equilibration, the rings were pre-constructed with 1 × 10^−6^ M phenylephrine (PE) until the stable contractile curve was attained (5–8 min), and the vasorelaxant effect of *R. vesicarius* was determined using a cumulative dosing approach [29,30].

### 2.13. Calcium Channel Blocking Activity

*R. vesicarius* was assessed against persistent contractions induced by high-K^+^ (80 mM) in isolated tissue preparation of rabbit aorta. These contractions were conciliated by activating voltage-dependent Ca^++^ channels, which caused a contractile response by initiating extracellular Ca^++^ influx. Substances that inhibit Ca^++^ ion influx through these channels can ease contractions induced by high-K. Test material must be administered additively against sustained spasm generated by the high-K [31]. The test drug’s relaxant impact on spastic contractions is expressed as a percent (%) of the control contraction response.

### 2.14. Adrenaline Caused Platelet Adhesion

The blood was collected from the vein (marginal ear) of the rabbit and centrifuged at 3000 rpm for 15 min to extract platelet-rich plasma. ADR (2 µM) was administered as a supplement to the samples. Impedance aggregometry was used to determine platelet adhesion, whereas flow cytometry was performed to assess platelet activation before and after supplementation [32]. The impact of three different ADR doses on platelet adhesion was initially investigated (*n* = 5). Following that, platelet adhesion was determined following ADR treatment and *R. vesicarius* at 50, 100, and 150 µg/mL (*n* = 5).

### 2.15. Statistical Analysis

The mean SEM of the ex vivo values for vasorelaxant activity was used. Sigmoidal dose-response curves (non-linear regression) were plotted, and EC_50_ values (with 95 percent CI) were obtained using the software Graph Pad Prism version-8 (Graph Pad Software, San Diego, CA, USA) (variable slope). In vivo data were assessed using a one-way analysis of variance (ANOVA) and Dunnett’s multiple comparison test. Statistical significance was defined as * *p* ≤ 0.05, ** *p* ≤ 0.001, and *** *p* ≤ 0.0001.

## 3. Results

### 3.1. Phytochemical Analysis

The presence of saponins, phenols, tannins, flavonoids, anthraquinones, and coumarins was confirmed by phytochemical analysis by visually monitoring the extract’s pre-specified color shift and precipitate development, while alkaloids were not detected (Table 1).

### 3.2. HPLC Analysis

Numerous phytoconstituents were validated in varied quantities using HPLC analysis; among them, the indispensable phytochemicals, kaempferol, Gallic acid, catechin, coumarin, *p*-coumaric acid, ferulic acid, emodine, rutin and quercetin were found based on retention time (Figure 1).

### 3.3. DPPH Assay

**Figure 2:**
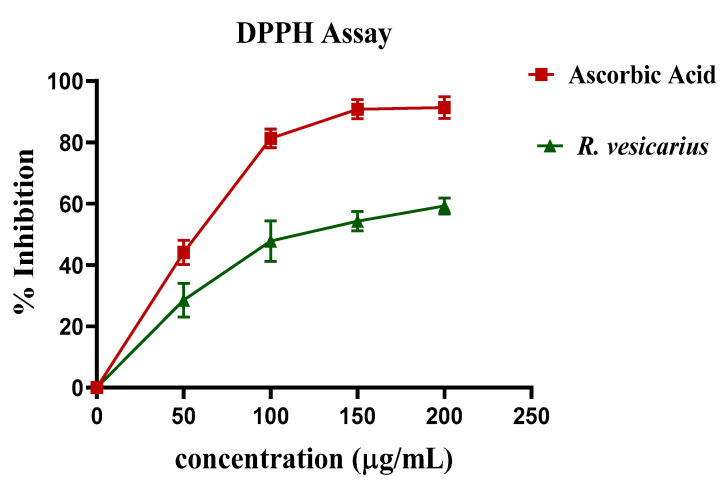
Antioxidant potential of *R. vesicarius* concerning ascorbic acid by DPPH assay (*n* = 5).

In the DPPH assay, the leaf extract of *R. vesicarius* (aqueous-methanolic) exhibited significant antioxidant activity at a 200 µg/mL concentration of approximately ascorbic acid (Figure 2).

### 3.4. Nitric Oxide Radical Scavenging Assay

**Figure 3:**
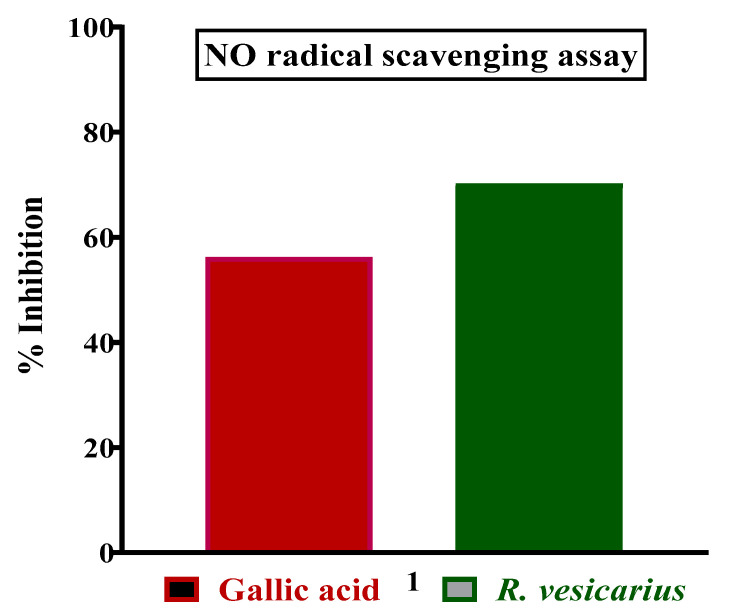
Nitric oxide scavenging potential (%) of aqueous-methanolic leaf extract of *R. vesicarius.*

*R. vesicarius* shown the antioxidant activity by competing with oxygen to scavenge for the nitrite radical produced by nitoprusside at physiological pH in an aqueous setting. Concentration dependent increase in antioxidant activity was noticed until it reached a plateau. *R. vesicarius* had shown, at 77.2%, the highest free radical scavenging activity in comparison with standard Gallic acid, as shown in Figure 3.

### 3.5. Evaluation of Myocardial Infarction

**Figure 4:**
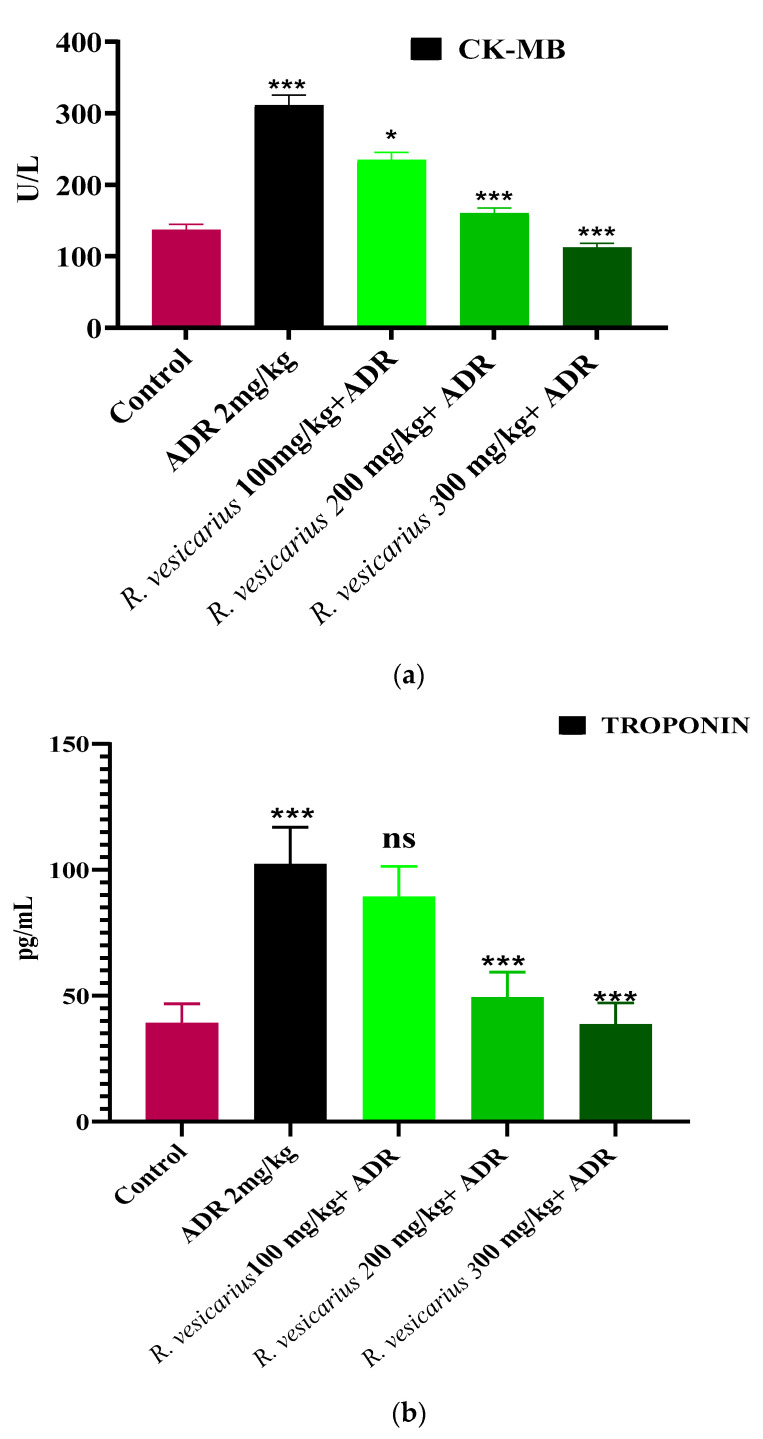

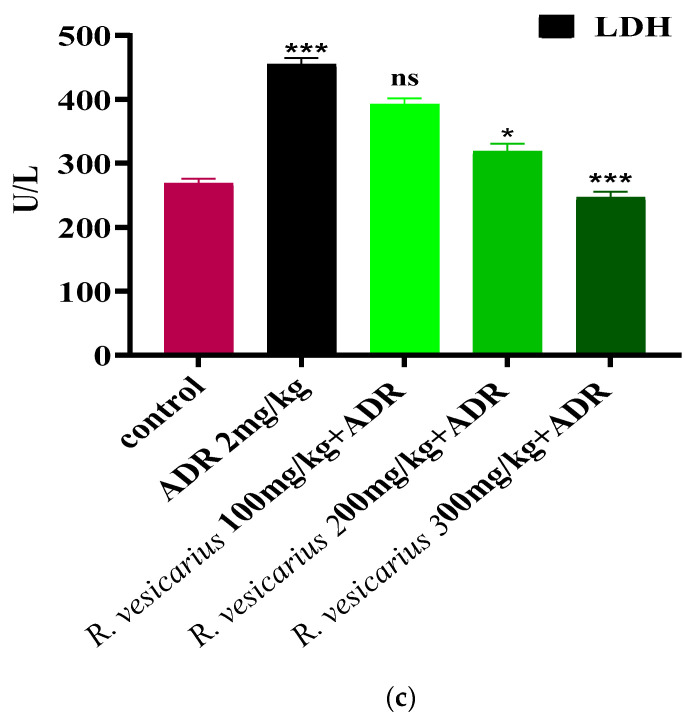
Cardioprotective impact of *R. vesicarius* extract on the cardiac markers, LDH (**a**), CK-MB (**b**), and troponin (**c**) in the presence of ADR. ANOVA with one-way analysis of variance and Dunnett’s multiple comparison test was used (* *p* ≤ 0.005, *** *p* ≥ 0.001, and ns *p* ≤ 0.005, *n* = 5).

**Figure 5:**
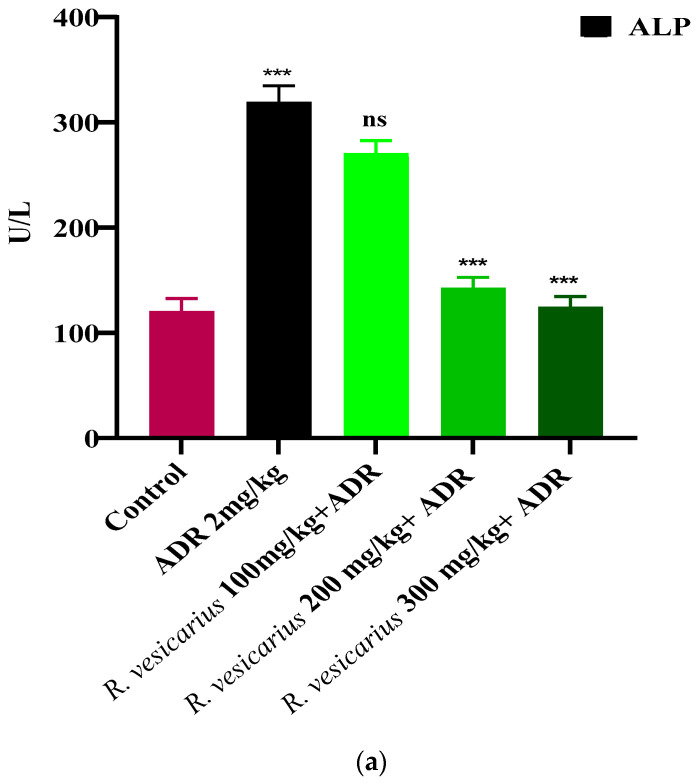

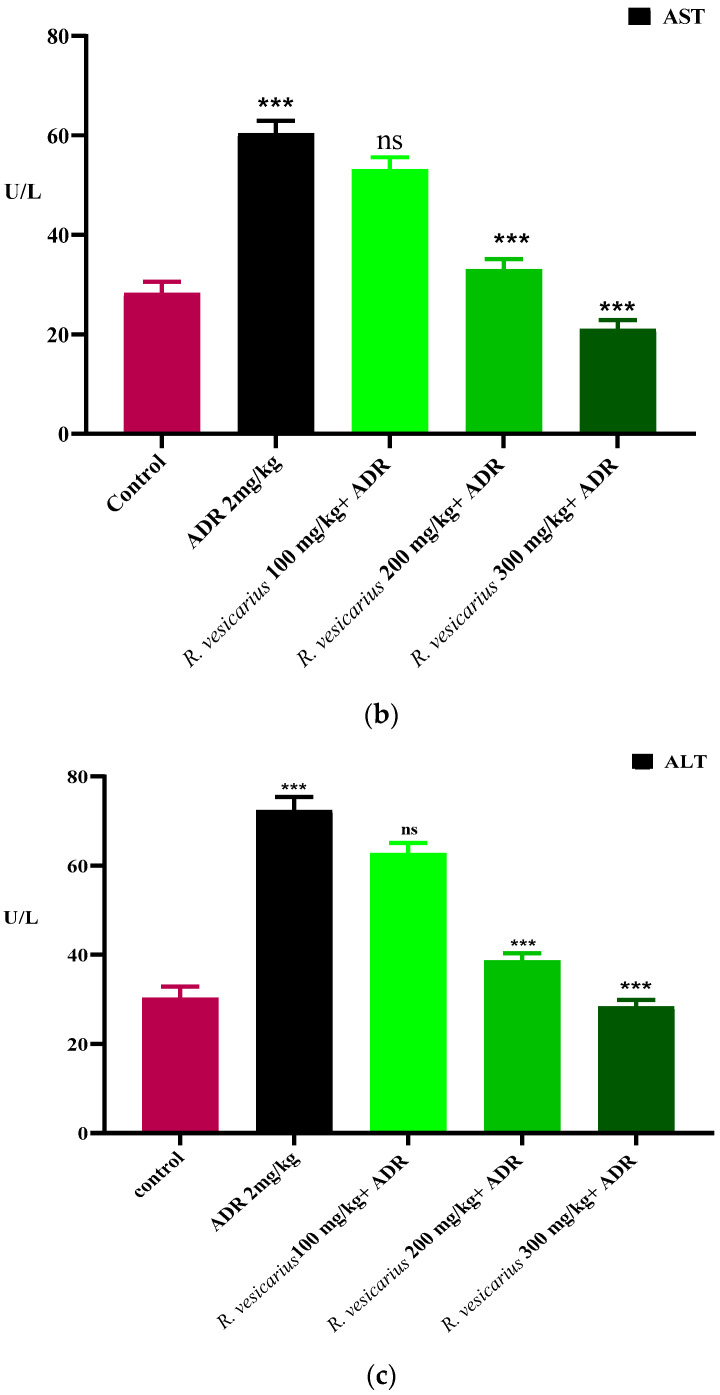

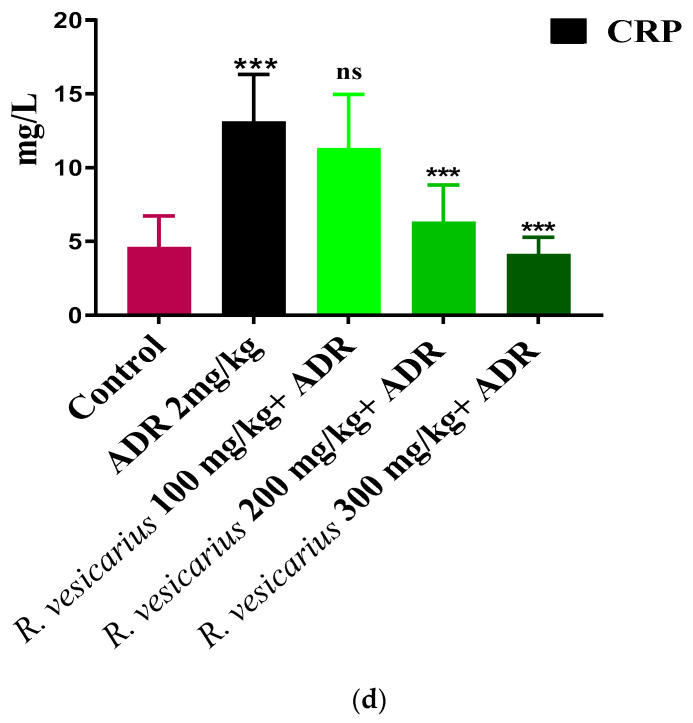
Cardioprotective impact of *R. vesicarius* extract on cardio-hepatic markers ALP (**a**), AST (**b**), ALT (**c**), and CRP (**d**) in the presence of ADR. ANOVA with one-way analysis of variance and Dunnett’s multiple comparison test was used (*** *p* ≥ 0.001, and ns *p* ≤ 0.005, *n* = 5).

ADR significantly increases cardiac markers (CK-MB, LDH, and troponin) and cardio-hepatic biomarkers (CRP, ALP, ALT, and AST) in comparison to control (*p* ≤ 0.05). The ADR-induced MI rabbits (Group 2) displayed dramatically elevated cardiac markers. In contrast, the groups (four and five) were treated with *R. vesicarius* at doses of 200 and 300 mg/kg b.w., and exhibited dose-dependent resistance to ADR-induced cardiac injury. Group 3 was statistically insignificant when 100 mg/kg body weight was used. In comparison to the ADR-intoxicated group, Groups 4 and 5 receiving *R. vesicarius* leaf extract showed significantly decreased average AST, troponin, ALP, CK-MB, ALT, LDH, and CRP levels (*p* = 0.05) (Figure 4 and Figure 5).

#### Effect on Heart to Body Weight Ratio

**Figure 6:**
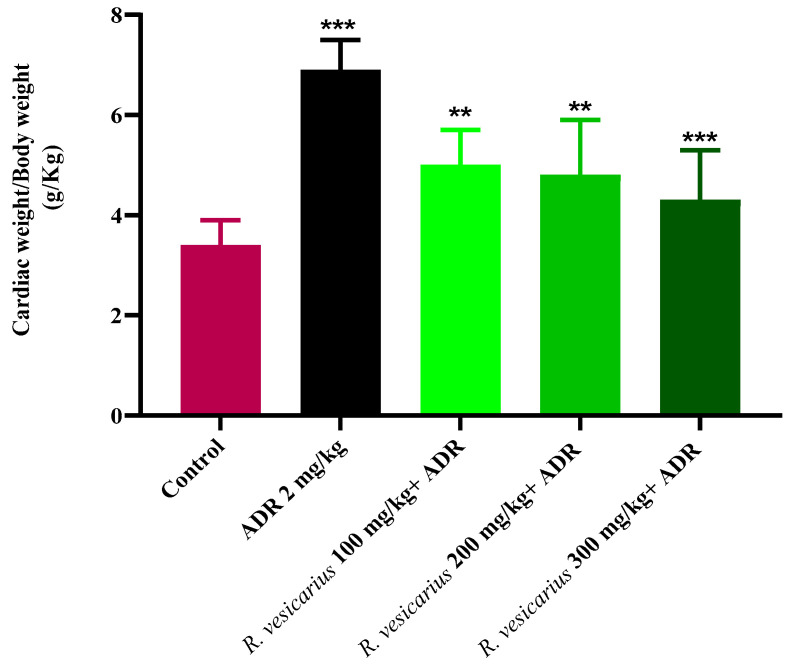
Cardioprotective impact of *R. vesicarius* extract on heart weight to body weight ratio against ADR-induced LVH and control. ANOVA with one-way analysis of variance and Dunnett’s multiple comparison test was used (** *p* ≤ 0.005, *** *p* ≥ 0.001, and *n* = 5).

ADR had a significant effect on the ratios compared to the control group. All three groups treated with *R. vesicarius* had significantly lower heart-to-body weight ratios than the ADR-treated group (Figure 6).

### 3.6. Histopathology

**Figure 7:**
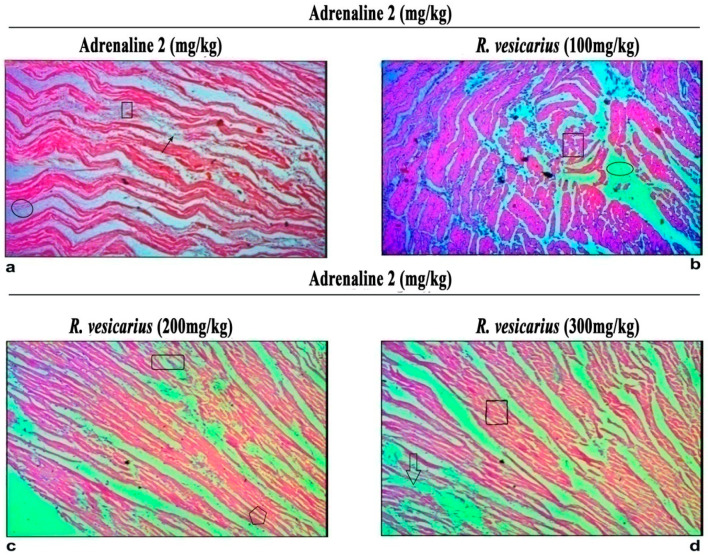
Histopathological changes in the ventricle part of rabbits’ hearts are depicted in this photomicrograph (**a**,**b**) *R. vesicarius* 100 mg/kg + ADR (**c**) 200 mg/kg *R. vesicarius* (**d**) 300 mg/kg *R. vesicarius* + ADR In comparison to the ADR-treated group, less inflammatory cells, cardiomyocyte deterioration, infiltration, and fibrosis were noticed in a dose-dependent manner; rectangular shapes indicate extended cardiomyocytes, upward arrows depict cellular infiltration, oval shapes depict cardiac fibrosis, and downward arrows indicate a dense normal cluster of cardiomyocytes. Magnification factor of 100.

Heart tissue fragments from the ADR-induced LVH group demonstrated a significant difference in cardiac cell architecture. Interstitial edema, shredding of muscle fibers, cellular infiltration, vacuolar disintegration, mottled staining, capillary expansion, hemorrhage, and myocardium blockage were all observed histologically in the ADR-induced LVH group. While all groups had extensive necrotic lesions, those treated with *R. vesicarius* had reduced myocardial degeneration (Figure 7).

### 3.7. Vasodilator Activity

**Figure 8:**
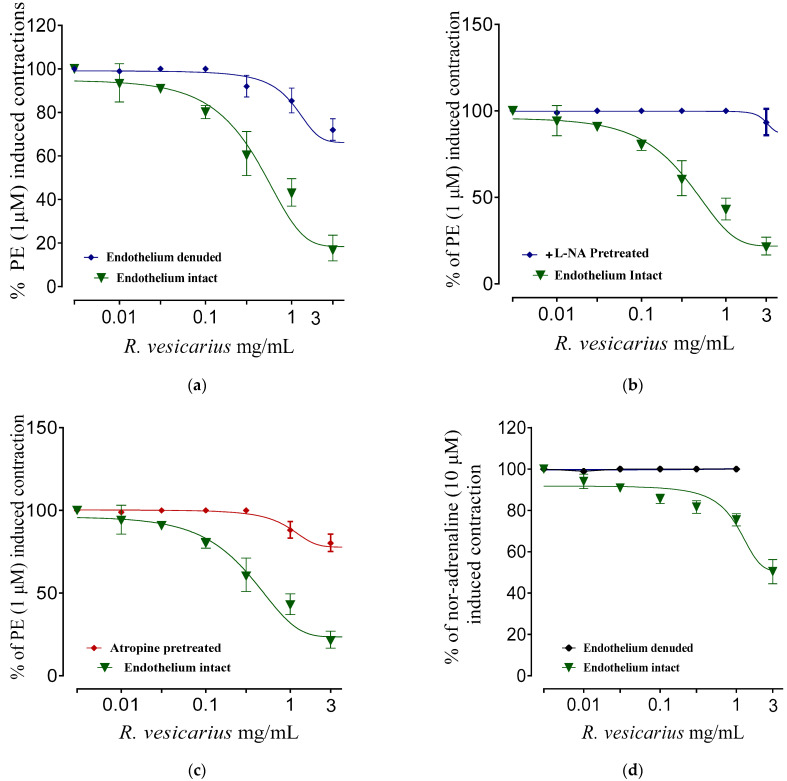
Dose–response curves of *R. vesicarius* extract against PE (1 μM) caused contraction for endothelium intact and denuded rabbit aorta (**a**) pretreated with L-NA (1 × 10^−4^ M) (**b**) Pretreated with atropine (1 μM) (**c**) against nor-adrenaline (10 μM) induced contraction endothelium intact and denuded rabbit aorta (**d**). The values (*n* = 5) are shown as mean ± SEM.

The isolated rabbit aorta rings showed endothelium-dependent partial vasodilation against phenylephrine (PE) (1 μM) causing spastic contraction (Figure 8a). Likewise, *R. vesicarius* showed the vasodilatory effect of varying strengths when pretreated with L-NA and atropine (1 M) (Figure 8b,c). Similarly, *R. vesicarius* showed almost 50% vasodilatory effect against noradrenaline (10 μM) caused vasoconstriction, suggesting multiple pathways’ involvement (Figure 8a–d).

### 3.8. Calcium Channel Blocking Activity

**Figure 9:**
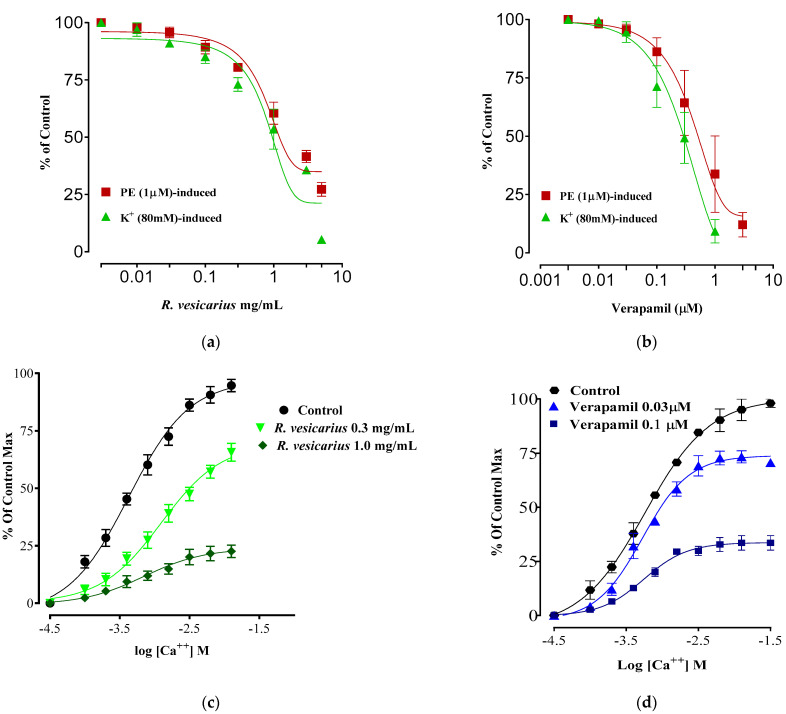
Dose–response curves of R. vesicarius extract (**a**) and verapamil (**b**) against PE (1 mM) and K^+^ (80 mM) induced contractions, their respective effect on the sigmoidal dose–response curves of CaCl_2_ in isolated rabbit aorta preparations (**c**,**d**). The values (*n* = 5) depicted are mean ± SEM.

*R. vesicarius* had a more significant vasodilatory impact against K^+^ (80 mM) generated contractions than PE (1 M) induced contractions, which was identical to verapamil (standard calcium channel blocker). The sigmoidal dose–response curves of CaCl_2_ in isolated rabbit aorta preparations showed a similar pattern (Figure 9).

### 3.9. Antiplatelet Aggregatory Effect

**Figure 10:**
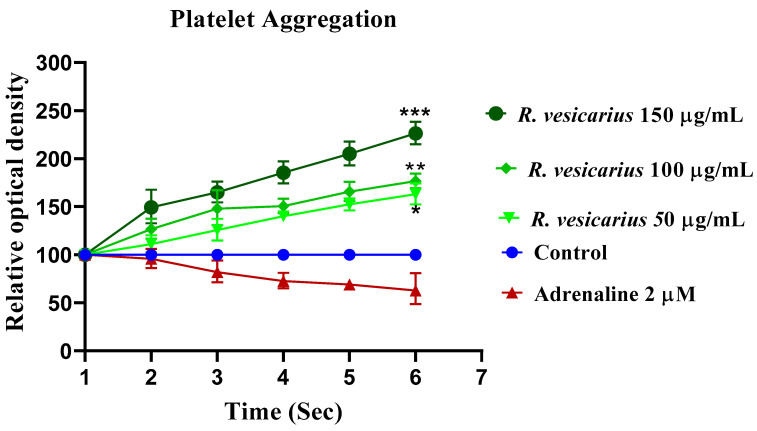
Antiaggregatory effect of *R. vesicarius* extract against ADR caused adhesion on human platelet. All three doses of *R. vesicarius* showed significant antiaggregatory impact in a dose-dependent manner (* *p* ≤ 0.005, ** *p* ≥ 0.002, *** *p* ≥ 0.000 and *n* = 5).

When ADR (2 µM) was added to a suspension of washed human platelets, the optical density at 600 nm decreased significantly, depicting platelet adhesion. The platelet adhesion was seen at 37 °C. In a dose-dependent pattern, *R. vesicarius* (50, 100, and 150 µg/mL) reduced platelet aggregation (Figure 10).

### 3.10. Acute Oral Toxicity Dose Test

*R. vesicarius* extract was found safe at doses up to 6000 mg/kg b.w. in an acute oral dose toxicity test. No fatality or illness was seen in any animal subjected to any dose.

## 4. Discussion

CVDs, notably MI, IHD, and cardiac hypertrophy, are the significant basis of mortality and morbidity worldwide [2]. Plants are commonly utilized to treat various cardiovascular problems, from mild to severe. The objective of this study was to test *R. vesicarius* leaf extract, which is used extensively in South Asia to treat cardiovascular illnesses without supporting pharmacological data [14], and is a wild edible herb with well-known medicinal properties [15,18,19]. Several medicinal plant therapies have been used to treat cardiac disorders from ancient times. However, no scientific data has been reviewed and published on the molecular mechanism behind *R. vesicarius’* cardioprotective effect using cellular and molecular techniques. This article will discuss how *R. vesicarius,* a medicinal plant, demonstrates pharmacotherapeutic promise in vitro, in vivo, and ex vivo in animal experiments, affecting cardiac and vascular diseases. *R. vesicarius* offers cardioprotection by inhibiting, altering, and controlling the synthesis of glycoproteins, structural, contractile, and regulatory proteins; this traditional medicinal plant acts as a preventative and therapeutic agent. These proteins regulate calcium levels and aid in mitochondrial activity [1]. Cardioprotective properties of medicinal plants have been demonstrated by their ability to reduce damage to cardiomyocytes, monocytes, endothelial cells, and vascular smooth muscle cells [16]. The opening of the K_ATP_ channel demonstrated cardioprotective effects in cardiomyocytes, as well as increased secretion of atrial natriuretic peptide, cardiac hypertrophy, apoptosis, oxidative stress, inflammation suppression, and the endothelial nitric oxide synthase nitric oxide (NOSNO) signaling pathway, have all been identified as beneficial effects of the medicinal herbs [17]. HPLC study of *R. vesicarius* leaf extract (aqueous-methanolic) confirmed the presence of rutin, quercitrin, and emodin (Figure 1). Rutin is now a well-established cardioprotective compound that inhibits cardiovascular pathogenesis at cellular and molecular levels by downregulating the ERK1/2 pathway and has already been reported for; cardioprotection, antihypertensive, antioxidant, and thrombolytic effects [33], and quercitrin reduces cardiac dysfunction via AMPKα dependent pathway and already documented for; oxidative stress stabilizer, antithrombotic, vasorelaxant, cardioprotective, diuretic, and bronchodilator effects [34]. Similarly, emodin is reported for cardioprotection via downregulating the TNF-α, NF-κB, and caspase-3 signaling pathways and effectively produced; vasorelaxation, anti-inflammatory, antithrombotic, anti-myocardial hypertrophy, anti-oxidative damage, antihypertensive and diuretic effects [35]. Considering the cardioprotective potential of rutin, emodin, and quercitrin, which are well detected in the HPLC of *R. vesicarius* (Figure 1), it is logical to believe that these phytoconstituents will be responsible for its cardioprotective effect as well as the phytoconstituents that may complement each other against the oxidative stress caused by ADR.

ADR is a non-selective adrenoreceptor that has been well-documented in experimental animals to develop AMI and LVH at different doses [7]. ADR has been shown to raise the weight of the heart (LVH) at low doses as a result of oxidative stress through an increase in arterial pressure via positive ionotropic and chronotropic effects [8]. In comparison, ADR administered at significantly greater dosages for two consecutive days results in myocardial apoptosis, edema, necrosis, and endocardial injury, all of which contribute to AMI [8]. ADR significantly increased the levels of cardiac markers troponin, LDH, and CK-MB, as well as cardiohepatic biomarkers ALP, AST, CRP, and ALT (*p* = 0.001). In contrast, the three groups receiving the *R. vesicarius* leaf extract had significantly lower cardiac and cardiohepatic markers than the ADR-treated group, implying its cardioprotective properties (Figure 3 and Figure 4).

Compared to the ADR-treated group, the cardiac weight to body weight ratio was considerably lower in Groups 4 and 5 treated with *R. vesicarius* (Figure 5), indicating dose-dependent protection against LVH. The presence of flavonoids in plants has been attributed to their cardioprotective properties [36,37], and *R. vesicarius* extract was shown to be abundant in flavonoids (Table 1), [13]. Considering the cardioprotective effect of flavonoids, it may be considered on logical grounds that the cardioprotective function of *R. vesicarius* is based on flavonoids. Abridged levels of CRP were observed in all three groups treated with *R. vesicarius* extract compared to the ADR-intoxicated group, which may be due to the vitamin C or flavonoid-dependent antioxidant capacity (Figure 2). Our findings corroborate the antioxidant potential of *R. vesicarius* reported earlier [38,39]. Saponins have been shown to reduce lipid peroxidation levels dose-dependently [40]. The findings of this study corroborate earlier research on saponins/saponin-containing plant extracts’ cardioprotective properties [30,31] as *R. vesicarius* is found to be rich in saponins (Table 1).

Earlier research [20] revealed that *R. vesicarius* had a strong inhibitory impact on LPO, confirming its cardioprotective potential, as LPO is the primary contributing factor to ADR-induced MI [1,6].

Histopathology of the ventricular fraction of the ADR-intoxicated group showed extensive injury of cardiac macrocells capillary distention, myocardium obstruction, including muscle fiber disintegration, cellular infiltration, interstitial edema, vacuolar disintegration, mononucleate cellular infiltration, tear, hemorrhage, vacuolar disintegration, and interstitial edema (Figure 6). In Groups 4 and 5, *R. vesicarius* treatment decreased inflammatory cells and myocardial degeneration. In comparison, extensive necrotic lesions were found in the ADR-intoxicated group (Figure 6), correlating with the biochemical analysis results (Figure 3 and Figure 4), which support its utility as a cardioprotective drug.

ADR promotes Ca^++^ inflow by activating voltage-gated calcium channels in the heart or raising cardiomyocytes’ intracellular calcium reserves [5,7]. This increased Ca^++^ burden results in oxidative stress [1,5]. The larger dosage of ADR (2 mg/kg b.w.) results in aortic and coronary channel constriction [7]. PE (1 M) and K^+^ (80 mM) both produce hypertension by vasoconstriction, mostly via activation of the alpha 1-adrenergic receptor and/or opening of L-type calcium channels [31], both of which contribute to the accumulation of oxidative stress (Figure 7). The vasodilator effect of *R*. *vesicarius* may be due to its ability to block these voltage-gated L-type calcium channels (Figure 7), as vasorelaxation was noticed at a lower concentration in response to K^+^ (80 mM) induced contraction than in response to PE (1 M) induced contraction. The compounds that relax the K^+^ (80 mM) induced contraction are believed to be calcium channel blockers [1,5,6]. Similar to verapamil, a negative ionotropic and chronotropic response was seen during the ex vivo experiment (Figure 7). Numerous earlier investigations have demonstrated that flavonoid-dependent vasodilation occurs in plants [36,37]. The phytochemical analysis discovered a high flavonoid content in the *R. vesicarius* (Table 1, Figure 1), providing a strong basis to trust in its cardioprotective activity. Anticoagulant and antithrombotic medications are routinely prescribed to cardiovascular patients and are critical cardioprotection sources in contemporary pharmacology [41]. Simultaneously, ADR is a well-reputed procoagulant [7]. Platelet transfusion is carried out significantly by the ADR, possibly by activating the alpha-adrenergic receptors of platelets, and it has been well recognized in prior research that ADR significantly boosts arachidonic acid aggregation [42]. In patients with the acute coronary syndrome (ACS), dual antiplatelet therapy (DAPT) with salicylic acid (SA) and an adenosine diphosphate (ADP) receptor antagonist like prasugrel, clopidogrel, ticagrelor, etc., decreases the risk of thrombosis compared to SA alone. However, it is also linked to enhancing the risk of sudden and perioperative bleeding [43]. When ticagrelor, a third-generation ADP-receptor antagonist, is administered instead of clopidogrel, efficacy and bleeding risk are increased. If bleeding occurs or emergency surgery is required, the effective platelet suppression in patients receiving continued ticagrelor medication becomes a substantial disadvantage. As a result, a high rate of serious perioperative bleeding problems has been documented in ACS patients undergoing or who have recently completed DAPT with SA and ticagrelor. Perioperative bleeding risk in cardiac patients receiving or recently discontinuing ADP receptor antagonist medication associated with residual ADP-dependent platelet aggregability. Even minor changes in residual platelet aggregability were found to influence bleeding risk [44]. The data indicate that *R.vesicarius* decreased ADR-induced activation and aggregation at all three doses tested. Thus, *R. vesicarius* has been used in CVD patients to treat and prevent severe bleeding in patients receiving ticagrelor, and SA. *R. vesicarius* has significantly reduced ADR’s platelet adhesion (Figure 8), contributing another section to the book on its utility in cardiovascular illnesses.

## 5. Conclusions

*Rumex vesicarius* has been shown to have antioxidant, hypotensive, vasodilatory, calcium channel blocking, and anticoagulant properties. The cardioprotective activity of *R. vesicarius* aqueous-methanolic leaf extract might be attributed to the variety of phytoconstituents found in the plant. *R. vesicarius* treatment may restore antioxidants in cardiomyocytes, which are required for resilience to the oxidative dent caused by ADR. However, an accurate molecular pathway of cardioprotection has yet to be found. An LC-MS/MS spectrum is indicated for further characterization. Additionally, cardiac glycosides, flavonoids, anthraquinones, and tannins were discovered during phytochemical screening, which may influence cardiovascular diseases, notably hypertension-induced LVH and MI. Finally, *R. vesicarius’* therapeutic utility in cardiovascular illnesses has been established in in vivo, in vitro, and ex vivo research. It will open the door for creating novel drugs to treat cardiovascular diseases by influencing numerous pathways.

## Figures and Tables

**Table 1:** Phytochemical analysis of *R. vesicarius* aqueous- methanolic leaf extract.

Tests	Observation	Results
Alkaloid	No PPT	−
Phenols	Light purple colour	+
Tannins	Light purple colour	+
Coumarins	Yellow fluorescence	+
Saponins	1 cm froth	+
Anthraquinones	Pink colour	+
Flavonoid	Light yellow colour	+

PPT = Precipitate formation, froth = Presence of froth in test tube. + = present, − = absent.

## Data Availability

Not applicable.

## Sample Availability

Samples of the compounds are not available from the authors.
